# Exploration of Social Engagement as a Risk Factor for Alzheimer's Disease among Indigenous Africans

**DOI:** 10.1002/alz70860_106892

**Published:** 2025-12-23

**Authors:** Joshua O Akinyemi, Carolina Scaramutti, Dingtian Cai, Farid Rajabli, Gabriel O Ogunde, Larry D Adams, Kara L Hamilton‐Nelson, Michael L Cuccaro, Daniel A. Dorfsman, Brian W Kunkle, Izri M Martinez, Andrew F Zaman, Fred Stephen Sarfo, Albert Kwaku Akpalu, Alfred K Njamnshi, Albertino Damasceno, Biniyam A Ayele, Yared Z Zewde, Thierry Adoukonou, Jean N Ikanga, Judith Boshe, David Ndetei, Reginald Obiako, Adefolakemi Ogundele, Kolawole Wahab, Godwin Osaigbovo, Paul Nwani, Damas Andrea Mlaki, Kamada Lwere, Paul Olowoyo, Olufisayo Oluyinka Elugbadebo, Mayowa Ogunronbi, Oyedunni Arulogun, Michelle Nichols, Stella Maria Paddick, Temitope Hannah Farombi, Njideka U Okubadejo, Richard Walker, Olusegun Baiyewu, Mayowa O Owolabi, Goldie S Byrd, Jeffery M Vance, Christiane Reitz, William S Bush, Jonathan L Haines, Raj N Kalaria, Margaret Pericak‐Vance, Rufus O. Akinyemi, Azizi A Seixas, Adesola Ogunniyi, African Dementia Consortium

**Affiliations:** ^1^ College of Medicine, University of Ibadan, Ibadan, Oyo, Nigeria; ^2^ Department of Psychiatry and Behavioral Sciences, University of Miami Miller School of Medicine, Miami, FL, USA; ^3^ John P. Hussman Institute for Human Genomics, University of Miami Miller School of Medicine, Miami, FL, USA; ^4^ Dr. John T. Macdonald Foundation Department of Human Genetics, University of Miami Miller School of Medicine, Miami, FL, USA; ^5^ Institute for Advanced Medical Research and Training, College of Medicine, University of Ibadan, Ibadan, Oyo, Nigeria; ^6^ University of Miami Miller School of Medicine, Miami, FL, USA; ^7^ Kwame Nkrumah University of Science and Technology, Kumasi, Ghana, Kumasi, Ghana; ^8^ University of Ghana, Accra, Ghana; ^9^ Brain Research Africa Initiative, Yaounde, Centre, Cameroon, Yaounde, Centre, Cameroon, Cameroon; ^10^ Faculty of Medicine, Eduardo Mondlane University, Maputo, Mozambique; ^11^ College of Health Sciences, Addis Ababa University, Addis Ababa, Ethiopia; ^12^ University Teaching Hospital, Department of Neurology, Parakou, Benin; ^13^ Emory University, Atlanta, GA, USA; ^14^ University of Kinshasa, Kinshasa, Kinshasa, Congo (The Democratic Republic of the); ^15^ Kilimanjaro Christian Medical University College, Moshi, Tanzania, United Republic of; ^16^ Africa Institute of Mental and Brain Health, Nairobi, Kenya; ^17^ University of Nairobi, Nairobi, Kenya; ^18^ Neurology Unit, Department of Medicine, Ahmadu Bello University/Ahmadu Bello University Teaching Hospital, Zaria, Nigeria; ^19^ Psychogeriatric Unit, Neuropsychiatric Hospital, Aro, Abeokuta, Nigeria, Abeokuta, Abeokuta, Nigeria; ^20^ Department of Medicine, University of Ilorin, Ilorin, Nigeria; ^21^ Jos University Teaching Hospital, Jos, Plateau, Nigeria; ^22^ Nnamdi Azikiwe University Teaching Hospital, Anambra State, Nigeria, Nnewi, Anambra, Nigeria; ^23^ Mirembe National Mental Health Hospital, Dodoma, Dodoma, Tanzania, United Republic of; ^24^ Makerere University, Kampala, Uganda, Uganda; ^25^ Ekiti State University Teaching Hospital, Ekiti, Ekiti, Nigeria; ^26^ University College Hospital, Ibadan, Oyo, Nigeria; ^27^ University of Ibadan, Ibadan, Oyo, Nigeria; ^28^ University of South Carolina, Charleston, SC, USA; ^29^ Newcastle University, Newcastle, North East England, United Kingdom; ^30^ University College Hospital, Ibadan, Nigeria; ^31^ Global Brain Health Inistitute, Trinity College Dublin, Dublin, Dublin, Ireland; ^32^ College of Medicine, University of Lagos, Lagos, Nigeria; ^33^ Northumbria Healthcare NHS Foundation Trust, North Shields, United Kingdom; ^34^ Translational and Clinical Research Institute, Newcastle University, Newcastle, Newcastle Upon Tyne, United Kingdom; ^35^ University College Hospital, Ibadan, Oyo State, Nigeria; ^36^ Department of Psychiatry, University of Ibadan, Ibadan, Oyo, Nigeria; ^37^ College of Medicine, University of Ibadan, Ibadan, Oyo State, Nigeria; ^38^ Wake Forest University School of Medicine, Winston‐Salem, NC, USA; ^39^ Department of Neurology, University of Miami Miller School of Medicine, Miami, FL, USA; ^40^ The Taub Institute for Research on Alzheimer's Disease and The Aging Brain, Columbia University, New York, NY, USA; ^41^ Department of Population and Quantitative Health Sciences, Institute for Computational Biology, Case Western Reserve University, Cleveland, OH, USA; ^42^ Department of Epidemiology and Biostatistics, Case Western Reserve University, Cleveland, OH, USA; ^43^ Newcastle University, Newcastle, United Kingdom; ^44^ John P. Hussman Institute for Human Genomics, University of Miami, Miami, FL, USA; ^45^ Department of Informatics and Health Science Data, University of Miami Miller School of Medicine, Miami, FL, USA

## Abstract

**Background:**

Social engagement (SE) is increasingly recognized as key modifiable risk factor for Alzheimer's Disease (AD), yet its role among indigenous African populations is less explored. Understanding how SE influences AD risks, progression and treatment is crucial for developing effective population‐specific interventions. We investigated the association between SE, and AD among indigenous Africans from nine countries.

**Method:**

Data were collected by the African Dementia Consortium as part of the ongoing READD‐ADSP study. We analysed a sample of 1,018 participants (509 case‐control pairs matched by age, sex and ethnicity). Cases had AD while controls were cognitively unimpaired. SE was assessed through self‐reported frequency of interactions with close individuals, categorized as low (<2 times/week) and high (≥5 times/week). Additionally, radio and television listening habits were recorded. Conditional logistic regression was used to assess associations, adjusting for education, living arrangement, urban/rural residence, religion and socioeconomic status.

**Result:**

Mean age was comparable between cases (75.6 ± 9.5 years) and controls (75.3 ± 9.5 years, *p* = 0.613). Childhood SE did not differ significantly (*p* = 0.445) but in adulthood, SE was lower among cases (27.0%) than controls (20.5%). Low SE in adulthood was significantly associated with higher odds of AD (AOR=1.89, 95% CI: 1.33‐2.70). More controls (57.7%) than cases (31.8%) listened to radio almost daily, which was linked to reduced odds of AD (AOR=0.29, 95% CI: 0.21‐0.43). Watching TV daily was associated with slightly reduced odds (OR=0.69, 95% CI: 0.49‐0.99).

**Conclusion:**

Findings suggest that low SE is associated with AD risk in African populations, highlighting the need for socially inclusive interventions to promote social participation and reduce isolation. Future longitudinal research should explore protective factors and underlying mechanisms in diverse African contexts.

